# Obesity and Type 2 Diabetes: Adiposopathy as a Triggering Factor and Therapeutic Options

**DOI:** 10.3390/molecules28073094

**Published:** 2023-03-30

**Authors:** Angelica Artasensi, Angelica Mazzolari, Alessandro Pedretti, Giulio Vistoli, Laura Fumagalli

**Affiliations:** Department of Pharmaceutical Sciences, Università degli Studi di Milano, 20133 Milano, Italy; angelica.artasensi@unimi.it (A.A.); angelica.mazzolari@unimi.it (A.M.); alessandro.pedretti@unimi.it (A.P.); giulio.vistoli@unimi.it (G.V.)

**Keywords:** type 2 diabetes mellitus, diabesity, obesity, adiposopathy, insulin resistance

## Abstract

Obesity and type 2 diabetes (T2DM) are major public health concerns associated with serious morbidity and increased mortality. Both obesity and T2DM are strongly associated with adiposopathy, a term that describes the pathophysiological changes of the adipose tissue. In this review, we have highlighted adipose tissue dysfunction as a major factor in the etiology of these conditions since it promotes chronic inflammation, dysregulated glucose homeostasis, and impaired adipogenesis, leading to the accumulation of ectopic fat and insulin resistance. This dysfunctional state can be effectively ameliorated by the loss of at least 15% of body weight, that is correlated with better glycemic control, decreased likelihood of cardiometabolic disease, and an improvement in overall quality of life. Weight loss can be achieved through lifestyle modifications (healthy diet, regular physical activity) and pharmacotherapy. In this review, we summarized different effective management strategies to address weight loss, such as bariatric surgery and several classes of drugs, namely metformin, GLP-1 receptor agonists, amylin analogs, and SGLT2 inhibitors. These drugs act by targeting various mechanisms involved in the pathophysiology of obesity and T2DM, and they have been shown to induce significant weight loss and improve glycemic control in obese individuals with T2DM.

## 1. Background

Nowadays, the role of insulin and its influences are continuously gaining attention since metabolic disorders have reached global pandemic proportions. In fact, insulin resistance (IR) is clinically associated with several metabolic disorders, including glucose intolerance, dyslipidemia, hyperuricemia, and hypertension. Hence, there is an urgent need to establish the mechanisms of IR and possible pharmacological treatments for these metabolic conditions. 

Beside being correlated to different metabolic syndromes, IR is also one of the major underlying mechanisms responsible for type 2 diabetes mellitus (T2DM), which consists of an array of multiple and complex disorders characterized by hyperglycemia and resulting from the combination of different metabolic and homeostatic disturbances that are sustained over time. Despite significant investments in clinical care, research, and public health interventions, T2DM is on the rise, and there does not appear to be any sign of a reduction in the rate of increase. Nearly 90% of the reported 537 million cases of diabetes worldwide suffer from T2DM, and these numbers are expected to rise to 783 million by 2045 [1]. Indeed, with the growing demand for more effective antidiabetic drugs, medicinal chemistry has made significant synthetic efforts towards the development of new drugs [2,3,4]. The main goal is to develop molecules that can specifically target systems involved in the metabolism of glucose, such as hepatic glucose synthesis, insulin signaling pathways, and glucose transporters. New understandings regarding the pathophysiological mechanisms of T2DM and diabetes led to the identification of new targets, which increased the development of new single-target antidiabetic drugs. Nonetheless, the potential of multi-target compounds has also been explored to treat these metabolic diseases [5,6].

As just mentioned, IR characterized the pathophysiology of T2DM, which results in early hyperinsulinemia and a steady loss in the ability of pancreatic cells to make insulin. Prolonged suboptimal glycemic control increases the risk of both microvascular (i.e., retinopathy, neuropathy, and nephropathy) and macrovascular complications (i.e., cardiovascular disease). However, despite being recognized as a complex disease, T2DM is diagnosed solely on persistently elevated levels of glycemia or glycated hemoglobin (HbA_1c_).

In this scenario, in order to develop new drugs for the treatment of this chronic disease, diabetes-related research has been largely focused on glycemic values and vascular complications. These goals have been major challenges since the early 90s, so much so that the combined progress of public health measures (i.e., awareness about modifiable risk factors and improved access to interventions) and the availability of new therapies contributed to the reduction of rates of major lower limb amputations and mortality from vascular causes [7]. 

Notwithstanding the encouraging outcomes, newer lines of evidence point to a shift in management trends and the rise of obesity as a novel treatment target.

Obesity is a metabolic disease and seems to be correlated with the rising burden of T2DM [8,9]. As a matter of fact, more than 80% of people who suffer from T2DM are classified as overweight or obese [10]. These two pathologies are deeply interconnected since they share many pathophysiological mechanisms, including IR, ectopic adiposity, inflammation, and β-cell dysfunction.

### Adiposity Impact on T2DM

**Figure 1:**
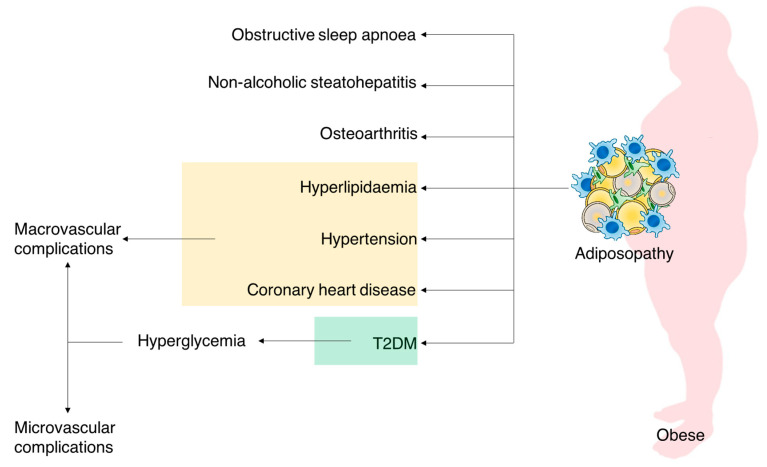
Illustration of the wide-ranging effects of adiposopathy.

Several studies support the benefit of a sustained loss (at least 15%) of body weight, which exerts major benefits on T2DM-related endpoints and cardiovascular events [11]. Changing modifiable risk factors, such as diet and lifestyle, is useful for treating obesity and preventing diabetes; however, for some people, these modifications are hard to maintain over time. Besides the improvement of these behaviors, anti-obesity pharmacotherapies are already available on the market. They usually provide benefit through appetite suppression or/and by inhibiting caloric intake, although management of the diabetic phenotype while reducing the number of side effects is still an unreached goal. In-depth characterization of targets and pathways involved in these complex, multifaceted diseases is an essential aim for the development of effective pharmacological agents. Thus, a promising opportunity for drug discovery relies on innovative targets and mechanisms of action that are now underrated. An emerging therapeutic framework is to target the adipose tissue since obesity and T2DM share a deep connection to abnormal adiposity (Figure 1), also known as adisopathy [12].

**Figure 2:**
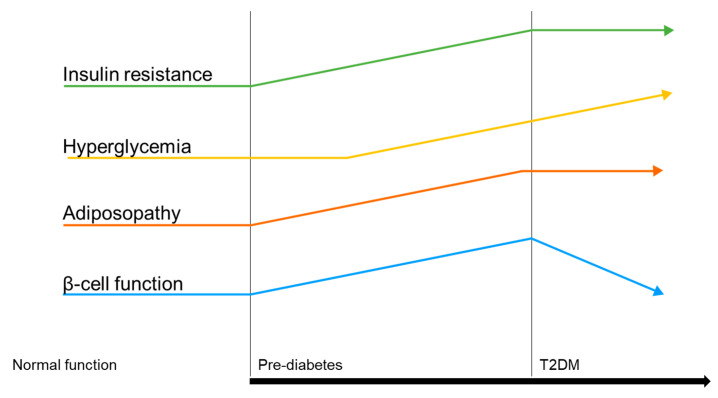
Illustration of the pathophysiological disease drivers in T2DM.

In more detail, white adipose tissue (WAT) is a remarkably complex organ that plays a significant role as a key energy reservoir. However, in obese and diabetic subjects, WAT often becomes highly dysfunctional, leading to adipocyte hypertrophy, visceral adiposity, and ectopic fat deposition, which result in the development of cardiometabolic syndromes [13]. Adiposity is also deeply linked to compensatory insulin secretion. This hypersecretion leads to insulin resistance and eventually to β-cell failure and results in a shift from the pre-diabetes stage to T2DM. As a matter of fact, adiposity and insulin resistance are strong predictors for full-blown T2DM, as represented in Figure 2.

Interestingly, one of the hallmarks of obesity-linked inflammation in WAT is the increased number of macrophages, which are also involved in the development of IR [14]. In addition, among obese individuals, alterations in the polarization of macrophages are possible, which indicates changes in the innate immune system concomitant with the metabolic syndrome. Unhealthy WAT expansion is accompanied by a shift in the adipokine secretory profile, which typically includes an increase in the pro-inflammatory factors, such as TNF, interleukin-1β (IL-1β), IL-6, IL-8, and leptin, with a concomitant reduction in the anti-inflammatory factors, such as IL-10 and adiponectin. The exact molecular mechanism of this increased inflammatory state in IR is not completely understood, but it is definitely multifactorial. Besides this, dysfunctional WAT may impact glucose homeostasis via other pathways that are not directly related to inflammation: for example, macrophages contribute to lipid homeostasis and to desensitize WAT to insulin [15,16]. As a result, dysfunctional adipose tissue has been linked to several clinical scenarios, namely fibrosis, hypoxia, and mitochondrial dysfunction [17].

Besides the strong relationship between insulin resistance and obesity [18], weight reduction always provides an advantage, whether the pathophysiology of T2DM is dominated by insulin resistance or β-cell dysfunction. Losing weight may also lead to improved metabolic and glycemic control, minimizing the overall treatment burden.

Therefore, a weight-centric, or even better, adipose-centric intervention would slow the disease course and benefit other associated cardiovascular risk factors, preventing long-term microvascular and macrovascular complications of T2DM [5,11]. 

## 2. Weight Loss Interventions

### 2.1. Overview

Determining the degree to which an individual is overweight or obese is essential to applying the most suitable guidelines for obesity management. However, the use of BMI-based criteria to determine eligibility for weight loss interventions is flawed because it excludes a significant percentage of the T2DM population from treatment, particularly those whose ethnic or racial backgrounds are more likely to develop metabolic comorbidities despite having a BMI below the obesity threshold [19]. In every case, successful strategies to facilitate substantial weight loss involved multicomponent interventions combining lifestyle modifications and pharmacotherapy. However, long-term maintenance of weight loss is challenging. Weight loss that is induced by dieting often results in an early rapid weight loss over 6 months, followed by a plateau and progressive regain over 1–3 years. The underlying reasons are unclear, although a potential explanation may lie in the several physiological changes intrinsic to obesity, such as the appetite dysregulation in the brain’ subcortical areas, which results in an increased drive to eat and a reduction of energy expenditure [20]. This is particularly accentuated when environmental conditions contribute to obesity (e.g., poor physical activity, easy access to high-caloric food).

### 2.2. Bariatric Surgery

Another effective opportunity lies in bariatric surgery, also known as metabolic surgery. The growing consensus that bariatric surgery is an enduring treatment for obese patients is reflected in the rise in the number of procedures. Moreover, bariatric surgery is supported as an anti-diabetic intervention for people with T2DM and obesity by the International Diabetes Federation and the American Diabetes Association [21,22]. Indeed, multiple studies demonstrated the efficacy of bariatric surgery in improving glucose homeostasis and reducing the need for glucose-lowering medications [23]. As a matter of fact, weight-loss surgery also leads to improvements in micro- and macro-comorbidities of T2DM, namely hypertension and concentrations of triglycerides, LDL, and HDL cholesterol. These effects are induced by alterations in gastrointestinal hormone secretory patterns that control appetite, which impact eating habits through the gut-brain axis and may also directly reduce blood sugar levels [21]. Bariatric surgery has a significant potential to lead a T2DM subject into remission, which is defined as a normal HbA_1c_ value without glucose-lowering medications for at least three months [20,21]. This is due to the combination between weight loss-dependent and weight loss-independent effects on glucose metabolism [24,25]. Additionally, people who undergo bariatric surgery have a lower risk of being diagnosed with T2DM 15 years after their procedure than patients who do not [26].

The four most common bariatric operations performed worldwide are laparoscopic sleeve gastrectomy, laparoscopic Roux-en-Y gastric bypass, laparoscopic adjustable gastric banding, and duodenal switch. Among these surgeries, new research indicates that the bypass technique may result in better long-lasting weight reduction and glycemic management, while the sleeve procedure is linked to fewer reoperations [27]. However, these are all invasive procedures and are not without risks. Some of the most common adverse effects of bariatric surgery include postoperative surgical complications, micronutrient deficiencies, gastro-esophageal reflux (for sleeve gastrectomy), hernias and ulcers (for gastric bypass), and slippage and pouch dilatation (for adjustable gastric banding).

### 2.3. Pharmacotherapy for Chronic Weight Management

Until now, there are no available pharmacotherapies that are able to replicate such results; still, several agents have received regulatory authorities’ approval for chronic weight management (Table 1). Currently, only three are approved for chronic weight management: orlistat (Xenical^®^) Montgomery, AL, USA, liraglutide (Saxenda^®^) Bagsvaerd, Denmark, and semaglutide (Wegovy^®^) Bagsværd, Denmark. Orlistat is an inhibitor of gastrointestinal lipase, and it is able to reduce the absorption of ingested fat by approximately 30% [28]. As a result, the undigested fat is eliminated in the stool, which can lead to side effects such as stomach cramps, diarrhea, and flatulence. While the last two are GLP1 receptor agonists (GLP1RA), which work by decreasing appetite and increasing feelings of fullness. They are a synthetic version of GLP1, a hormone released by the body after eating. By mimicking GLP1 and binding to its receptors in the brain, they elicit the above mentioned beneficial effects. They also slow the emptying of the stomach, which can also prolong the feeling of fullness [29]. These drugs are usually prescribed for individuals with a body mass index (BMI) of 30 or more, or for individuals with a BMI of 27 or more who also have other health conditions such as diabetes or high blood pressure. Furthermore, the FDA has approved some combination therapies for obesity treatment, such as phentermine/topiramate (Qsymia^®^) Campbell, CA, USA [30] and bupropion/naltrexone (Mysimba^®^) San Diego, CA, USA [31]. The former one is a combination of an appetite suppressant (phentermine) and an anti-seizure medication that also affects appetite and satiety to help with weight loss (topiramate). While the latter is a combination of two drugs that have been FDA-approved for the treatment of depression (bupropion) and addiction (naltrexone).

## 3. Management of T2DM and Obesity

Besides these obesity pharmacotherapies, several glucose-lowering agents that are FDA-approved for the treatment of T2DM have been recognized to promote weight loss (Table 2). These include biguanides (metformin), GLP1R agonists, amylin analogs, and sodium-glucose cotransporter 2 (SGLT2) inhibitors [37].

### 3.1. Biguanides

**Figure 3:**
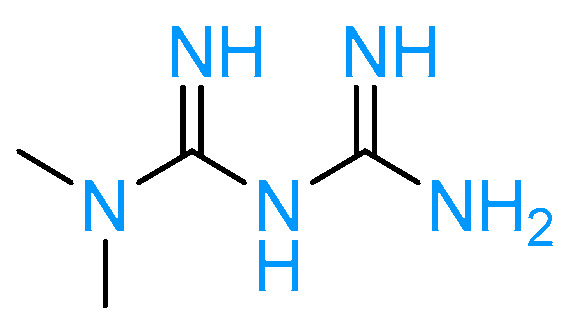
Metformin structure.

Metformin, represented in Figure 3, is the sole FDA-licensed antihyperglycemic medication in this pharmacological family, and its introduction drastically improved T2DM management. It helps to lower blood sugar levels by increasing insulin sensitivity and reducing the amount of glucose produced by the liver. Although metformin was introduced into clinical use notwithstanding its cellular mechanisms, over the last few years its complex modes of action have been better defined.

Metformin works by inhibiting mitochondrial glycerophosphate dehydrogenase, indirectly activating adenosine monophosphate-activated protein kinase (AMPK), and lowering cytosolic dihydroxyacetone phosphate while increasing the cytosolic NADH/NAD ratio [24]. As a result of its high levels in the intestine, metformin increases glucose metabolism through glycolysis, and an excessive amount of lactate is produced in intestinal epithelial cells. Lactate is then converted to glucose in hepatocytes, creating an ineffective intestinal-liver cycle that increases energy expenditure [25]. Metformin accumulation in the gastrointestinal tract can affect not just epithelial brush border metabolism but also the altered microbiota composition of patients with T2DM, resulting in decreased serum lipopolysaccharides (LPS) levels, reduced inflammation, and improved insulin sensitivity [26].

Studies also show that metformin promotes weight loss and decreases food intake. This is justified by the AMPK-dependent effect in the brain, where metformin suppresses orexigenic peptides, neuropeptide Y, and agouti-related proteins, while the decrease in food intake is due to an increase in the expression of the leptin receptor gene in the arcuate nucleus to reduce central leptin sensitivity [27].

Additionally, chronical metformin treatment increases growth differentiation factor 15 (GDF15) and GLP1 levels, two key mediators of metformin-induced weight loss [27,28]. Metformin has also been shown to improve body composition in T2DM patients since it reduces visceral fat mass and abdominal subcutaneous fat [29]. Unfortunately, even though metformin may lead to some weight loss, the amount lost is far less than the amount desired. 

According to the Diabetes Prevention Study (DPP), which is the largest study to show the weight benefits of metformin, the average weight reduction after one year on the medicine is only 2.1 kg [30]. In 2 years of follow-up, the level of weight loss was substantially associated with adherence, with highly adherent patients experiencing an average 3.5% reduction in body mass, while low adherence was associated with weight-neutral status. Waist circumference was similarly influenced, with a lower weight circumference being correlated with the degree of adherence.

Given the safety and tolerability of metformin, as well as its mild weight-loss effect, the FDA has not approved metformin as a weight-loss agent. Nevertheless, it is currently and commonly used off-label in individuals who are at high risk for metabolic problems and who cannot tolerate alternative therapies. As a matter of fact, the 2016 American Association of Clinical Endocrinologists (AACE) guidelines on obesity management recommend the use of metformin (as well as acarbose and thiazolidinediones) in obese patients with evidence of prediabetes or insulin intolerance that does not respond to lifestyle medications or other anti-obesity medications (grade A; BEL 1) [31].

### 3.2. GLP1 Agonist

Incretin-based therapies are a prominent approach to successfully managing obesity and metabolic disorders such as T2DM. Patients who suffer from T2DM fail to achieve glycemic control due to a desensitization of incretin receptors [38,39,40]. Nevertheless, preclinical and clinical investigations demonstrate that glycemic control can be accomplished by medications [41,42] or weight reduction [43].

Incretin hormones include GLP1 and the glucose-dependent insulinotropic polypeptide hormone (GIP). These enteroendocrine hormones are released from the gut in response to intraluminal carbohydrates and fats [6].

They act mainly on the pancreas, where they exert their insulinotropic activity. Concomitantly, they play a direct role in several biological pathways involved in T2DM and obesity, such as satiety and lipolytic activity [44]. The numerous physiological effects are due to the fact that incretin receptors are G protein-coupled receptors expressed in many tissues, including the pancreas, brain, and gut. As a consequence, incretin hormones are interesting targets because not only do they act on different mechanisms involved in the pathophysiology, but they are also essential regulators of food intake and body weight. More in detail, one of the primary actions of incretin hormones is to stimulate insulin secretion from the pancreatic β-cells in a glucose-dependent manner, which means that they only activate insulin production when blood glucose levels are elevated. In addition to its effects on glucose homeostasis, incretin effects include slowing gastric emptying and promoting satiety, which helps reduce food intake and body weight [45].

GLP1 also exerts several cardiovascular effects, including improving endothelial function and reducing blood pressure. Moreover, GLP1 inhibits glucagon production from pancreatic α-cells, which helps lower hepatic glucose production and enhance peripheral glucose absorption [46]. The net effect of these actions is lower blood glucose levels.

Unlike GLP1, GIP may stimulate glucagon secretion at lower glucose levels and promote lipid synthesis and storage in adipose tissue, which can increase fat accumulation and body weight gain [47]. This has led to controversy about the potential role of GIP in the development of drugs for obesity and related metabolic disorders.

However, the administration of GIP receptor (GIPR) agonists (both central and peripheral) is reported to lead to a reduction in caloric intake and body weight [48,49]. The reason may lie in the fact that solely GIP receptors (GIPR) are expressed in WAT [50], and GIPR agonism has been reported to improve the ability of adipocytes to acutely clear dietary triglycerides by directly activating GIPR on adipocytes, indirectly via the lipogenic action of insulin, or through the combination of both [51]. GIP also promotes the healthy expansion of WAT, which decreases ectopic fat accumulation in tissues such as the heart, skeletal muscle, pancreas, and liver [52]. In addition, GIP also reduces proinflammatory immune cell infiltration [51,53], a process that is characterized by unhealthy WAT.

**Figure 4:**
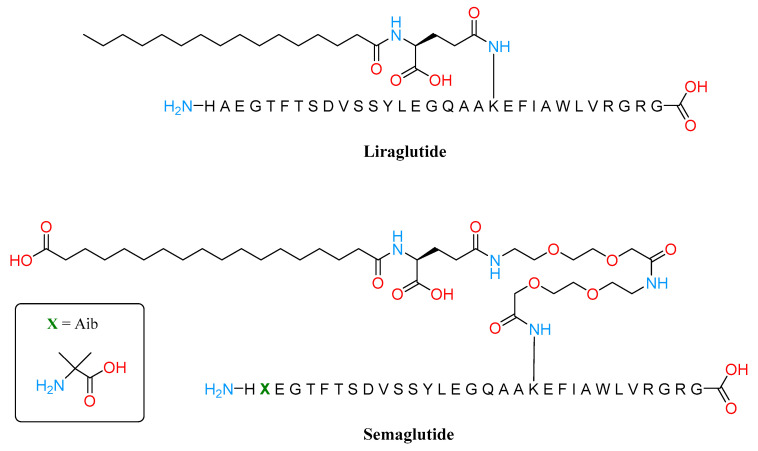
Structures of Liraglutide and Semaglutide.

Single or multitarget agents that replicate the effects of GLP1 and GIP are particularly appealing in T2DM due to the improvement of glucose homeostasis combined with weight-loss action and benefit adipose tissue health. Several GLP1RAs are approved for the treatment of T2DM in both Europe and the United States, such as exenatide (Byetta^®^) San Diego, CA, USA, albiglutide (Tanzeum^®^) Middlesex, UL, USA, dulaglutide (Trulicity^®^) Indianapolis, IN, USA, and lixisenatide (Adlyxin^®^) Paris, France. Furthermore, as already mentioned, among the approved GLP1RAs, some drugs, namely liraglutide and semaglutide (Figure 4), are also approved by the FDA and EMA as weight loss medications in individuals with a BMI of 30 or higher or a BMI of 27 or higher with a weight-related condition such as high blood pressure, T2DM, or high cholesterol.

Liraglutide and semaglutide are both long-acting GLP1RAs that exhibit pharmacodynamic effects for 24 h per day despite different dosing intervals and dosages. Liraglutide’s structure possesses minor sequence alterations from the parent peptide GLP1: the differences include an Arg in position 28, instead of a Lys, and in position 20, the Lys is covalently linked with a C16 acyl chain via a glutamoyl spacer.

The fatty acid facilitates the bond with albumin, allowing the GLP1 analog to decrease the renal clearance and extend its duration of action [54].

Clinical studies with liraglutide 1.8 mg have demonstrated weight reduction in people with T2DM of up to 3.6% (3.3 kg) at 26 weeks and 4.7% (5.0 kg) at 56 weeks [29,55,56], whereas trials with liraglutide 3.0 mg have demonstrated weight loss in patients with obesity of between 7.9% (8.9 kg) and 8.2% (7.3 kg) at 56 weeks [33,56,57].

Moreover, obese people can lose up to 12.5 kg in total during the run-in phase of a low-calorie diet, of which half was observed during the liraglutide 3.0 mg treatment period [58].

The indirect impact of improved glucose management on body weight homeostasis may be the cause of the difference in weight reduction between patients with T2DM and subjects with obesity.

Regarding semaglutide, the peptide backbone was modified at position 8, where an aminoisobutyric acid (Aib) was introduced instead of an Ala and an acetylated Lys26. Structure-activity investigations proved that C18 diacid together with a γGlu and two oligo (ethylen glycol) linkers resulted in the highest albumin affinity combined with GLP1R potency. Clinical studies demonstrated that semaglutide has a similar safety profile to other GLP1RAs while offering better weight reduction than liraglutide [59,60,61]. As a matter of fact, semaglutide causes an approximately two-fold larger weight loss than liraglutide and a three-fold greater weight loss than exenatide [59], even if they were not dose optimized in the same manner. Additionally, both liraglutide and semaglutide lower the cardiovascular risk in people with T2DM, a result that has not been seen with short-acting GLP1RAs [62].

**Figure 5:**
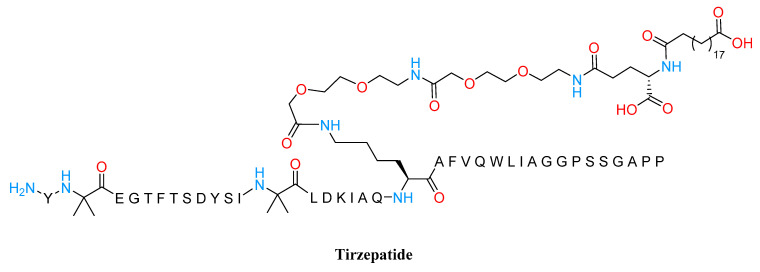
Structure of Tirzepatide.

Besides these GLP1RAs, dual GIP/GLP1RAs have been investigated as well, such as tirzepatide (LY3298176) [63], which is represented in Figure 5. This drug, based on the GIP sequence and attached to a C20 diacid moiety, was developed by Eli Lilly and approved by the FDA and EMA in 2022 for the treatment of adults with obesity or overweight with weight-related comorbidities [64]. This strategy extends its half-life to 5 days, making a once-weekly dosage possible. Tirzepatide showed in pre-clinical studies and clinical trials to strongly lower glucose and to exert weight reduction benefits with side effects that are equivalent to those of known GLP1 receptor agonists.

In the SURPASS phase 3 clinical trial program, 5, 10, and 15 mg of Tirzepatide have been investigated for patients suffering from T2DM. All three doses led to a larger reduction in body weight (from 8.2% up to 11.9% for the highest dose) and an improved HbA_1c_ value (2–2.3% reduction) than did 1.0 mg of semaglutide (i.e., −6.1% bodyweight and −1.9% HbA_1c_) over 40 weeks in addition to metformin [65].

The design of GLP1 and glucagon dual agonists has also been explored. This combination aims to take advantage of the satiety effect while balancing insulin secretion mediated by GLP1 and glucagon’s mobilization of hepatic glucose. The most promising compound is MEDI0832 (cotadutide), a synthetic peptide that results in side chain modification of the primary sequence of glucagon in order to confer GLP1R activity, in addition to an esterification with a palmitic fatty acid to facilitate albumin binding. This novel peptide is more potent toward the GLP1R, with a 3–4 times lower efficacy at the GLP1R compared with native GLP1, and an 8-fold lower potency at the GCGR (glucagon receptor) compared with natural glucagon. Cotadutide is under clinical development by AstraZeneca and is currently in Phase II of clinical studies [66]. Obese patients with T2DM who were on metformin monotherapy showed a mean reduction of body weight equal to 5% and a decrease in HbA_1c_ of 1.2% with a dose of 300 mg of cotadutide [67]. Table 3 represents several other multi-target drugs that are currently under development, such as BI 456906 (Boehringer Ingelheim, Ingelheim am Rhein, Germany), LY3305677 (Eli Lilly, Indianapolis, IN, USA) LY3437943 (Eli Lilly, Indianapolis, IN, USA USA), JNJ-54729518 (J&J, New Brunswick, NJ, USA), HM15211 (Hanmi, Seoul, Republic of Korea), NNC9204-1706 (Novo Nordisk, Bagsværd, Denmark), Alt-801 (Altimmune, Gaithersburg, Maryland, USA), and G3215 (Imperial College/Zihipp Ltd., London, United Kingdom). However, in general, this approach has not yielded satisfactory outcomes yet.

### 3.3. Amylin Analogs

**Figure 6:**
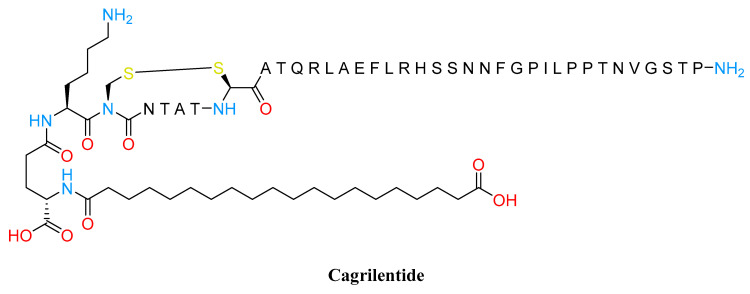
Cagrilentide structure.

Incretin hormones are only some of the released peptides from the gastrointestinal system in response to food intake. Among the plethora of satiety hormones, there is amylin (or islet amyloid polypeptide; IAPP), a 37-amino acid peptide, co-stored and co-secreted with insulin by pancreatic islet β-cells. IAPP relays signals to the hypothalamic nuclei and other areas of the subcortical areas of the brain, resulting in increased feelings of satiety and fullness. In addition, IAPP plays also a major role in glucose homeostasis by slowing gastric emptying, suppressing glucagon secretion, and exerting anorexigenic effects by working synergistically with leptin [75]. Furthermore, although the majority of licensed pharmacotherapies for weight control work on the hypothalamus to decrease hunger and energy intake, several preclinical studies demonstrate that amylin decreases weight by focusing on both the homoeostatic and hedonic areas of the brain [76,77]. Notably, in the obesity metabolic disturbance state, amylin secretion is increased leading to a desensitization of its receptor and even a reduction in its expression [78]. However, this situation can be recovered by improving plasma levels and gene expression of amylin mRNA and its receptor [75]. The synthetic amylin mimetic pramlintide is an approved treatment for diabetes (both type 1 and 2) mostly in combination with insulin. This drug promotes better glycemic control and modest but significant weight loss (after 16 weeks the weight loss is up to 3.7 kg after 120–240 µg of pramlintide three times a day) [79]. By contrast to pramlintide, cagrilintide is the first amylin analog to be investigated for weight management (Figure 6).

In a 26-week clinical study, 4.5 mg of cagrilintide (weekly dosed) achieved 7.8% mean weight loss versus placebo. In addition, the result obtained with 3.5 mg of cagrilintide was also greater than the 6.0% mean weight loss achieved with liraglutide at 3.0 mg [80]. 

Contrary to a placebo, weight loss with cagrilintide persisted for the full 26 weeks of therapy without hitting a plateau. Similarly to the outcomes with liraglutide at 3.0 mg, weight reductions following cagrilintide treatment were followed by an overall improvement in TFEQ (Three-Factor Eating Questionnaire) scores for cognitive restraint, emotional eating, and uncontrolled eating in the same trial. A further step along the way to identifying a compound able to manage weight and glycemic targets in people with T2DM has been to evaluate the combination between 2.4 mg of semaglutide (GLP1RA) and different doses of cagrilintide (1.2, 2.4, and 4.5 mg). The concomitant treatment of cagrilintide and semaglutide, in comparison to semaglutide alone, provided effective weight loss (15.4% up to 17.1%). Furthermore, glycemic control was improved regardless of the cagrilintide dose [81].

### 3.4. SGLT-2 Inhibitors

**Figure 7:**
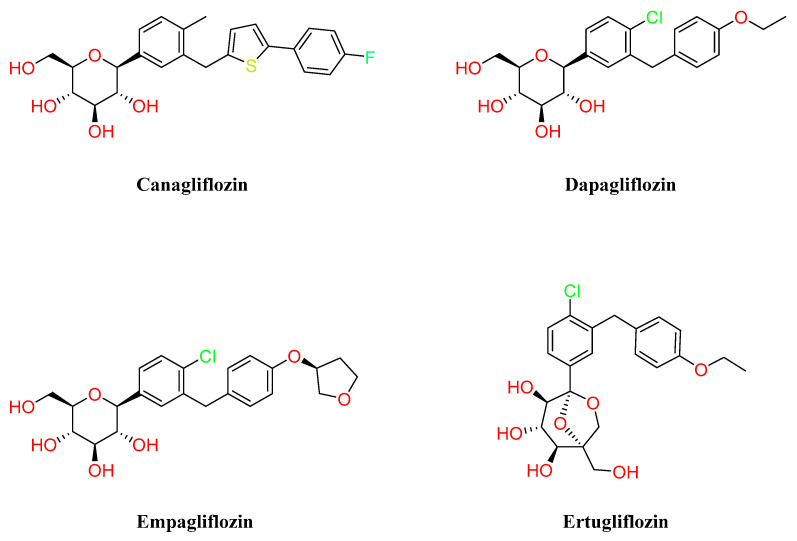
Structures of different SGLT-2 inhibitors.

Other glucose-lowering agents with weight-loss propriety are the sodium-glucose cotransporter 2 inhibitors (SGLT-2i). At the moment, there are several SGLT-2i available on the market namely, canagliflozin, dapagliflozin, empagliflozin, and ertugliflozin (Figure 7), or in development.

This class of small molecules is characterized by an insulin-independent mechanism and normalizes glycemia by avoiding the reuptake of filtered glucose in the kidney. In addition to the common primary outcome of glycemic control, this mechanism of action leads to further beneficial effects such as a reduction in blood pressure and weight [82,83]. Several clinical trials have been conducted on different SGLT-2i for the treatment of T2DM, and the decrease in weight occurs quickly within the first weeks of treatment, then it becomes more gradual [84]. The weight loss observed with SGLT-2 inhibitors is primarily caused by glycosuria, which leads to both energy and water loss via osmotic diuresis [85,86]. In T2DM patients treated with SGLT2i, 50 to 85 g of glucose are excreted daily, values close to those of healthy individuals that represent around 50% of the glucose-filtered load [87]. Interestingly, meta-analysis also points out a change in body composition: after 16 weeks on ipragliflozin, 50 to 70% of the total weight loss was from body fat, while 15 to 35% was from water weight [84,88,89]. In addition, a shift in metabolic processes was also demonstrated in T2DM subjects, where the inhibition of SGLT-2 is associated with increments in glucagon release and stimulation of lipid oxidation and lipolysis. Other physiological changes that could be responsible for weight loss include increased glucagon/insulin ratios that first cause the liver’s glycogen stores to be depleted and then activate gluconeogenesis using circulating amino acids, as well as a general switch from glucose to free fatty acids. The drop in amino acid levels eventually causes a change in mitochondrial morphology from fission to a sustained fusion state [84].

Furthermore, SGLT-2i reduces blood leptin, increases adiponectin levels, and shows cardiovascular protective properties, in particular in cases of heart failure, by reducing the adipose accumulation in the myocardium [90]. The degree of the cardiovascular benefit seems not to be related only to the weight loss but could include hemodynamic changes and the shift to ketone body metabolism [84]. SGLT-2i therapy has also been associated with improvements in renal and liver function in patients with T2DM [91,92].

Thus, pharmacotherapy with SGLT-2i is recommended for T2DM subjects who have indicators of high risk of renal or heart failure, although treatment with SGLT-2i is correlated with an increased risk of urinary and genital infections [93].

## 4. Conclusions

Obesity is a highly multifaceted chronic disease condition that poses a serious public health issue. Furthermore, it is associated with an increased risk of IR, T2DM, and several cardiovascular and chronic inflammatory diseases. As a matter of fact, obesity and T2DM are strictly interconnected since they share key pathophysiological mechanisms. Intensive dietary and lifestyle modifications can be effective for both obese and T2DM subjects by ameliorating glycemic control and producing weight loss. Such beneficial effects can also be achieved through existing pharmacological approaches that treat both obesity and T2DM and are already available on the market. When the weight loss, is at least 15% of the initial bodyweight, it can lead to significant improvement in the metabolic status and can also induce remission. However, better solutions are required for successful, significant, and long-lasting weight loss since maintaining a healthy weight is not so simple and there is a huge unmet need for effective pharmacotherapy with minimal side effects.

Nonetheless, it is important to underline that using BMI-based criteria to establish eligibility for weight loss interventions has several flaws. BMI is an inadequate indicator of a person’s health risks since it does not directly evaluate body fat mass or its potential for harm. Addressing obesity should focus on a more adiposopathy-based approach. Therefore, considering adiposity health should be essential, even if efforts are still needed to easily detect adipose tissue pathology. The progress toward personalized medicine in order to better define obesity and provide individualized treatment choices is a challenge to prioritize. Furthermore, it is also important to recognize that more efforts are needed to understand the complex link between adiposopathy, obesity, and T2DM in order to identify new possible targets and develop new effective active compounds while avoiding the rise of both obesity and T2DM.

## Figures and Tables

**Table 1:** Weight loss drugs.

Drug	Company	Dose, Administration	Approval	Mode of Action	Weight Loss (Placebo/Drug)
Orlistat (Xenical^®^)	Roche Pharmaceuticals	120 mg, TTD	1999–present (EU, USA)	Inhibitor of gastrointestinal lipase	−6.1% to −10.2% [32]
Liraglutide (Saxenda^®^)	Novo Nordisk	with titration 3.0 mg OD	2014–present (EU, USA)	GLP1R agonist	−2.6% to −8% [33]
Semaglutide (Wegovy^®^)	Novo Nordisk	2.4 mg, OW	2021–present (EU, USA)	GLP1R agonist	−2.4% to −14.9% [34]
Phentermine/ Topiramate (Qsymia^®^)	Vivus	with titration 15 mg/92 mg, OD	2012–present (USA)	Central norepinephrine release	−1.2% to −9.3% (dose-dependent) [30,35]
Bupropion/ Naltrexone (Mysimba^®^)	Orexigen Therapeutics	360 mg/32 mg, TD	2014–present (EU, USA)	Increased central norepinephrine and dopamine and opioid receptor antagonist	−1.3% to −6.1% (dose-dependent) [36]

TD, twice daily; TTD, three times daily; OD, once daily; OW, once weekly.

**Table 2:** Profiles of T2DM therapy.

	Metformin	DPP-IVi	GLP1RA	SGLT-2i	TZD	SU and GLN	Pramlintide
Hypoglycemia	Neutral	Neutral	Neutral	Neutral	Neutral	Mild/Moderate	Neutral
Weight	Slight Loss	Neutral	Loss	Loss	Gain	Gain	Loss
Renal/GU	Contraindicated if eGFR <30 mL/min/1.73 m^2^	Dose adjustment is necessary (except linagliptin) Effective in Reducing albuminuria	Exenatide not indicated CrCl <30	Not indicated for eGFR <45 mL/min/1.73 m^2^	Neutral	More Hypo Risk	Neutral
				Genital Mycotic infection			
			Possible benefit of Liraglutide	Possible benefit of Empagliflozin			
GI	Moderate	Neutral	Moderate	Neutral	Neutral	Neutral	Moderate
Cardiac	Neutral	Possible increase in hospitalization with alogliptin and saxagliptin	Liraglutide Prevents MACE events	Empagliflozin reduce CV mortality Canagliflozin reduce MACE events	Moderate risk for CHF	Possible ASCVD risk	Neutral
					May reduce stroke risk		
Bone	Neutral	Neutral	Neutral	Mild Fracture Risk	Neutral	Neutral	Neutral
Ketoacidosis	Neutral	Neutral	Neutral	DKA can occur in various Stress settings	Neutral	Neutral	Neutral

DPP-IVi, Dipeptidyl Peptidase-IV inhibitor; TZD, Thiazolidinedione; SU, Sulfonylurea; GLN, Glinide; GI, Gastrointestinal; GU, Genitourinary; eGFR, Estimated Glomerular Filtration Rate; CrCl, Creatinine Clearance; MACE, Major Adverse Cardiac Events; DKA, Diabetic ketoacidosis; CV, Cardiovascular; CHF, congestive heart failure; ASCVD, atherosclerotic 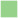 cardiovascular 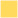
disease. 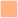
Possible benefit→Use with caution→Possible adverse effects.

**Table 3:** Current antidiabesity multitarget-drugs in development.

Drug	Company	Targets	Sequence Modified	Phase	Ref
Cotadutide	Altimmune	GLP1/GCGR	Glucagon	Phase II	[67]
BI 456906	Boehringer Ingelheim	GLP1/GCGR	Glucagon	Phase II	[68]
LY3305677	Eli Lilly	GLP1/GCGR	OXM	Phase I	[69]
LY3437943	Eli Lilly	GLP1/GIP/GCGR	-	Phase I	[70]
JNJ-54729518	J&J	GLP1/GCGR	OXM	Phase II	[71]
HM15211	Hanmi	GLP1/GIP/GCGR	Glucagon	Phase II	[72]
NNC9204-1706	Novo	GLP1/GIP/GCGR	-	Phase I	[73]
Alt-801	Altimmune	GLP1/GCGR	GLP1 andglucagon	Phase I	[74]
G3215	Imperial College/Zihipp Ltd.	GLP1/GCGR	OXM	Phase I	[71]
